# TRIM44 Is a Poor Prognostic Factor for Breast Cancer Patients as a Modulator of NF-κB Signaling

**DOI:** 10.3390/ijms18091931

**Published:** 2017-09-08

**Authors:** Hidetaka Kawabata, Kotaro Azuma, Kazuhiro Ikeda, Ikuko Sugitani, Keiichi Kinowaki, Takeshi Fujii, Akihiko Osaki, Toshiaki Saeki, Kuniko Horie-Inoue, Satoshi Inoue

**Affiliations:** 1 Department of Functional Biogerontology, Tokyo Metropolitan Institute of Gerontology, 35-2 Sakae-cho, Itabashi-ku, Tokyo 173-0015, Japan; kawabata2000@gmail.com (H.K.); azumak@tmig.or.jp (K.A.); 2 Division of Gene Regulation and Signal Transduction, Research Center for Genomic Medicine, Saitama Medical University, 1397-1 Yamane, Hidaka-shi, Saitama 350-1241, Japan; ikeda@saitama-med.ac.jp (K.I.); iku0215@saitama-med.ac.jp (I.S.); khorie07@saitama-med.ac.jp (K.H.-I.); 3 Department of Breast Oncology, Saitama Medical University International Medical Center, 1397-1 Yamane, Hidaka-shi, Saitama 350-1298, Japan; aosaki@saitama-med.ac.jp (A.O.); tsaeki@saitama-med.ac.jp (T.S.); 4 Department of Breast and Endocrine Surgery, Toranomon Hospital, 2-2-2 Toranomon, Minato-ku, Tokyo 105-8470, Japan; 5 Department of Pathology, Toranomon Hospital, 2-2-2 Toranomon, Minato-ku, Tokyo 105-8470, Japan; k.kinowaki@gmail.com (K.K.); tkshfj@gmail.com (T.F.)

**Keywords:** breast cancer, tripartite motif-containing 44 (TRIM44), nuclear factor kappa-B (NF-κB) signaling, cyclin-dependent kinase 19 (CDK19), matrix metallopeptidase 1 (MMP1)

## Abstract

Many of the tripartite motif (TRIM) proteins function as E3 ubiquitin ligases and are assumed to be involved in various events, including oncogenesis. In regard to tripartite motif-containing 44 (TRIM44), which is an atypical TRIM family protein lacking the RING finger domain, its pathophysiological significance in breast cancer remains unknown. We performed an immunohistochemical study of TRIM44 protein in clinical breast cancer tissues from 129 patients. The pathophysiological role of TRIM44 in breast cancer was assessed by modulating TRIM44 expression in MCF-7 and MDA-MB-231 breast cancer cells. TRIM44 strong immunoreactivity was significantly associated with nuclear grade (*p* = 0.033), distant disease-free survival (*p* = 0.031) and overall survival (*p* = 0.027). Multivariate analysis revealed that the TRIM44 status was an independent prognostic factor for distant disease-free survival (*p* = 0.005) and overall survival (*p* = 0.002) of patients. siRNA-mediated TRIM44 knockdown significantly decreased the proliferation of MCF-7 and MDA-MB-231 cells and inhibited the migration of MDA-MB-231 cells. Microarray analysis and qRT–PCR showed that TRIM44 knockdown upregulated *CDK19* and downregulated *MMP1* in MDA-MB-231 cells. Notably, TRIM44 knockdown impaired nuclear factor-kappa B (NF-κB)-mediated transcriptional activity stimulated by tumor necrosis factor α (TNFα). Moreover, TRIM44 knockdown substantially attenuated the TNFα-dependent phosphorylation of the p65 subunit of NF-κB and IκBα in both MCF-7 and MDA-MB-231 cells. TRIM44 would play a role in the progression of breast cancer by promoting cell proliferation and migration, as well as by enhancing NF-κB signaling.

## 1. Introduction

Breast cancer is the most common cancer in women worldwide, accounting for one-fourth of all cancers [1]. Systemic adjuvant treatment is a standard treatment strategy for breast cancer patients even without metastatic lesion, since breast cancer tends to metastasize from an early phase [2]. The clinical outcome of the patient is affected by the metastatic activity of the cancer cells and the effectiveness of the anti-cancer drugs, and therefore, enormous efforts have been made to identify diagnostic biomarkers that predict prognosis and effective treatment strategies [3].

Tripartite motif (TRIM) proteins typically have a structure containing RING-finger, B-box and coiled-coil domains [4]. So far, more than seventy kinds of proteins are found to have a similar domain structure, and they comprise the TRIM family [5,6,7]. Currently, accumulated evidence suggests that some of the TRIM-family proteins function as critical regulators for antiviral functions, immunity and carcinogenesis [8,9,10,11,12,13,14,15,16]. For example, we previously showed that TRIM25/Efp (estrogen-responsive finger protein) plays an oncogenic role in breast cancer by functioning as an E3 ubiquitin ligase that degrades cell cycle checkpoint 14-3-3σ [17].

TRIM44 is an atypical TRIM-family protein that lacks the RING-finger domain but contains a zinc-finger domain, which is often found in ubiquitin-specific proteases [8]. To date, several studies have reported that TRIM44 contributes to the progression of human malignancies such as lung cancer [18,19], esophagus and gastric cancers [20,21], breast cancer [21], hepatic cancer [22], and testicular germ cell tumor [23]. In terms of breast cancer, genomic amplifications or gains of TRIM44 were shown [21], but protein expression in breast cancer tissues or functions of TRIM44 in breast cancer cells were not evaluated in the report.

Here, we examined TRIM44 protein expression in breast cancer specimens by immunohistochemistry. The biological functions of this protein were also investigated in breast cancer cells.

## 2. Results

### 2.1. Immunostaining of TRIM44 in Human Breast Cancer

**Figure 1:**
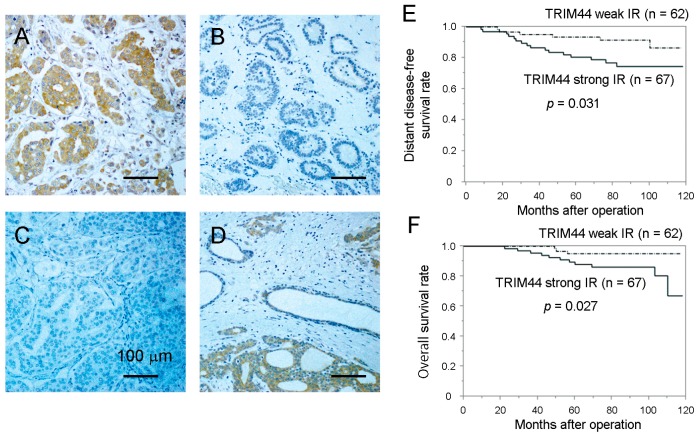
Relationship between TRIM44 immunoreactivity and prognosis of patients. (**A**) Representative micrograph of a breast cancer case with strong TRIM44 immunoreactivity (IR). TRIM44 was stained in the cytoplasm of cancer cells. The scale bar represents 100 μm. (**B**) Representative micrograph of a breast cancer case with negative TRIM44 IR. The scale bar represents 100 μm. (**C**) Representative micrograph of a breast cancer tissue applied non-specific rabbit IgG antibody as a negative control. The scale bar represents 100 μm. (**D**) Example of the tissue sample where TRIM44 IR was strong in the cancerous region and weak in the morphologically benign glands and stroma. The scale bar represents 100 μm. (**E**) Distant disease-free survival of breast cancer patients with strong or weak TRIM44 IR was shown by the Kaplan–Meier method. The solid line represents cases with strong TRIM44 IR, and the dashed line represents cases with weak TRIM44 IR. The statistical significance was evaluated using the log-rank test. (**F**) Overall survival of breast cancer patients with strong or weak TRIM44 IR was shown by the Kaplan–Meier method. The solid line represents cases with strong TRIM44 IR, and the dashed line represents cases with weak TRIM44 IR. The log-rank test was performed.

To explore the clinical value of TRIM44 expression in breast cancer, immunohistochemical analysis was performed with 129 patients who underwent surgical treatment of primary breast cancers. Most of them had estrogen receptor (ER)-positive primary tumors. Representative images for strong (Figure 1A), negative (Figure 1B) TRIM44 immunoreactivity (IR), and negative control (Figure 1C) are shown. Based on the IR criteria, 67 of 129 patients (52%) had tumors with strong TRIM44 IR, whereas the rest of the 62 patients had tumors with weak IR. Then, the relationship between TRIM44 IR and clinicopathological parameters was analyzed (Table 1). TRIM44 IR was significantly associated with nuclear grade (*p* = 0.033) and age (*p* = 0.044), but no significant association was observed between TRIM44 IR and other clinicopathological parameters examined, such as stage, pathological tumor size, lymph node status, ER status, progesterone receptor (PR) status and HER2 status.

### 2.2. Differential Expression Pattern of TRIM44 mRNA in Oncomine Database

The present immunohistological results of TRIM44 expression revealed that weak TRIM44 IR is often observed in non-cancerous regions. In contrast, we often observed strong IR in malignant regions in the same specimen (Figure 1D). Then, we explored whether TRIM44 mRNA expression could also be a biomarker for breast cancer. Based on the TCGA dataset in the Oncomine database (https://www.oncomine.org) for 389 invasive ductal breast carcinomas compared with 61 normal breast tissues, >1.5-fold higher expression of TRIM44 mRNA was shown in carcinomas versus normal tissues at a *p*-value of ~2 × 10^−10^ (Figure S1).

### 2.3. Relationship between TRIM44 IR and Patients’ Prognosis

We next examined the relationship between TRIM44 IR and the clinical prognosis of breast cancer patients. TRIM44 IR was significantly associated with a decreased incidence of distant disease-free survival (Figure 1E) and overall survival (Figure 1F) of patients. Univariate analysis of distant disease-free survival and overall survival using the Cox proportional hazard model demonstrated that TRIM44 IR could be a significant prognostic factor for distant disease-free survival and overall survival, in addition to the known prognostic factors such as tumor size, ER status and nuclear grade (Table 2). Multivariate analysis for 4 factors including tumor size, ER status, nuclear grade and TRIM44 IR showed that all of them are independent prognostic factors.

### 2.4. TRIM44 Knockdown Inhibits the Proliferation and Motility of Breast Cancer Cells

**Figure 2:**
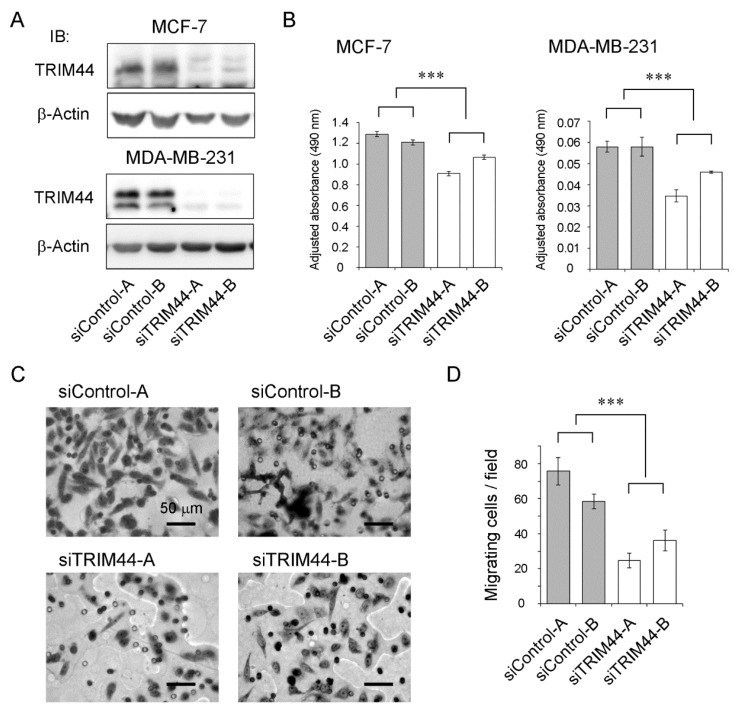
Effects of TRIM44 knockdown on proliferation and motility of breast cancer cells. (**A**) Western blot analysis of TRIM44 knockdown efficiency in MCF-7 and MDA-MB-231 cells. Two kinds of siRNAs for TRIM44 (siTRIM44-A and -B) as well as siRNAs not targeting human transcripts (siControl-A and -B) were used. β-Actin protein was blotted as an internal control. IB, immunoblot. (**B**) Inhibitory effect by siTRIM44 on the proliferation of MCF-7 and MDA-MB-231 cells. MTS assay was performed at day five after transfection of siRNAs. Results are expressed as mean ± SEM (*n* = 4). *** *p* < 0.001 (two-way ANOVA). (**C**) Inhibitory effect by siTRIM44 on the motility of MDA-MB-231 cells. Cells were incubated for 24 h after transfection of siRNAs, and migration during the next 24 h was evaluated. Cells on the lower side of the filters were stained by the Giemsa stain solution and visualized under microscope at a magnification of 400×. Representative photographs of migrating are shown. The scale bars indicate 50 μm. (**D**) Cells migrating to the lower surface of the filters were counted in five fields. Results are expressed as mean ± SEM (*n* = 5). *** *p* < 0.001 (two-way ANOVA).

In order to evaluate the biological functions of TRIM44 in human breast cancer cells, we transfected specific siRNAs for TRIM44 (siTRIM44-A/-B) in both ER-positive MCF-7 and ER-negative MDA-MB-231 breast cancer cell lines. Suppression of TRIM44 protein expression by TRIM44 siRNAs was confirmed by western blotting (Figure 2A). The effect of TRIM44 knockdown on cell proliferation was analyzed by an MTS assay. The proliferation of both MCF-7 and MDA-MB-231 cells was suppressed by TRIM44 knockdown (Figure 2B). The effect of TRIM44 on motility of MDA-MB-231 cells was evaluated by a transwell migration assay. The number of cells migrating through a filter with 8.0 μm pores in 24 h was evaluated (Figure 2C). The number of migrating cells treated with siTRIM44-A/-B was significantly lower than that of cells treated with control siRNAs (siControl-A/-B) (Figure 2D). In the case of MCF-7 cells, it was difficult to evaluate the effect of siTRIM44 due to low migration activity. These data indicated that TRIM44 could promote breast cancer cell proliferation and migration.

### 2.5. Augmented Nuclear Factor-Kappa B (NF-κB) Signaling by TRIM44

**Figure 3:**
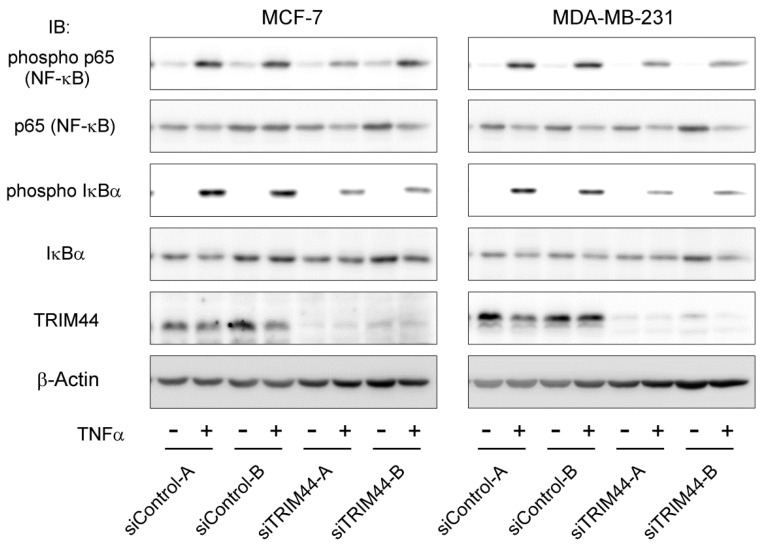
Effects of TRIM44 on the NF-κB signaling pathway in breast cancer cells. Knockdown of TRIM44 attenuated NF-κB signaling in breast cancer cell lines. Phosphorylation of the NF-κB p65 subunit and IκBα after TNFα treatment for 5 min was analyzed by western blotting. Transfection of siRNAs (2 nM) was performed by a reverse-transcription method 48 h before TNFα (10 mg/mL in MCF-7 and 20 mg/mL in MDA-MB-231) or vehicle (phosphate-buffered saline) treatment. Total and phosphorylated form-specific antibodies for NF-κB p65 and IκBα were used to evaluate phosphorylation of each protein. Attenuated phosphorylation of p65 and IκBα was observed in both MCF-7 and MDA-MB-231 breast cancer cells transfected with siTRIM44. Immunoblotting with TRIM44 antibody was performed to confirm knockdown of TRIM44. β-Actin protein was blotted as an internal control. IB, immunoblot.

The crosstalk between TRIM44 and the NF-κB signaling pathway has been reported in malignancies such as lung cancer cells [18] and hepatic cancer cells [22]. NF-κB has pathological relevance in malignancies, as it is known to protect cancer cells from apoptosis and to facilitate cell survival [24]. We thus evaluated the effects of TRIM44 on the NF-κB signaling pathway by western blot analysis and a luciferase reporter assay. TRIM44 knockdown caused attenuated phosphorylation of the p65 subunit of NF-κB and NF-κB inhibitor α (IκBα) on TNFα stimulation in both MCF-7 and MDA-MB-231 breast cancer cells (Figure 3). The reporter assay revealed TRIM44 knockdown significantly impaired NF-κB-mediated transcriptional activity stimulated by tumor necrosis factor α (TNFα) in both MCF-7 (Figure S2A) and MDA-MB-231 (Figure S2B) breast cancer cells. Elevated NF-κB-mediated transcriptional activity was observed in TRIM44 transfected breast cancer cells compared to mock transfected cells (Figure S2C,D). These data suggested that TRIM44 could enhance NF-κB signaling.

### 2.6. Identification of Target Genes for TRIM44

**Figure 4:**
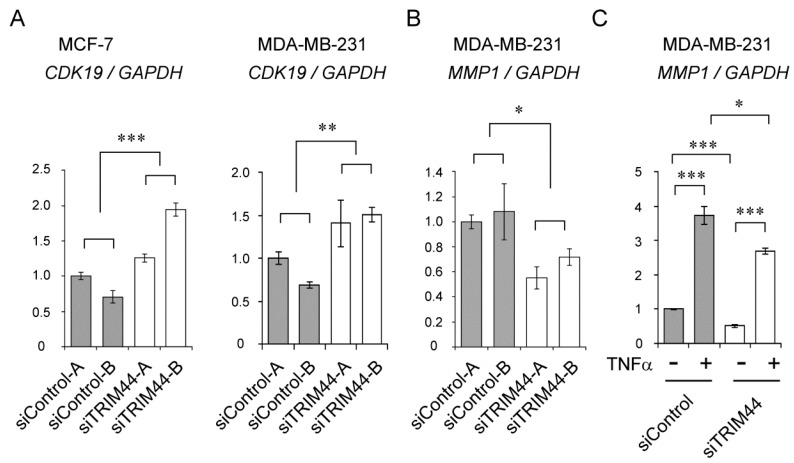
Regulation of *CDK19* and *MMP1* expression by TRIM44. (**A**) Knockdown of TRIM44 significantly increased *CDK19* mRNA expression in MCF-7 and MDA-MB-231 cells. siRNAs (10 nM) were transfected by the reverse-transcription method 42 h before harvesting cells. mRNA expression levels were normalized with GAPDH by the 2^−ΔΔ*C*t^ method. ** *p* < 0.01, *** *p* < 0.001 (two-way ANOVA). (**B**) Knockdown of TRIM44 significantly decreased *MMP1* mRNA expression in MDA-MB-231 cells. siRNAs (10 nM) were transfected by the reverse-transcription method 42 h before harvesting cells. mRNA expression levels were normalized with GAPDH by the 2^−ΔΔ*C*t^ method. * *p* < 0.05 (two-way ANOVA). (**C**) Expression of *MMP1* is induced by TNFα treatment. Transfection of siControl-B (siControl) and siTRIM44-B (siTRIM44) was performed by the reverse-transfection method at a concentration of 2 nM. 24 h after transfection, the cells were treated with TNFα (50 ng/mL) or vehicle (phosphate-buffered saline) for another 24 h before harvest. mRNA expression levels were normalized with *GAPDH* by the 2^−ΔΔ*C*t^ method. * *p* < 0.05, *** *p* < 0.001 (*t*-test).

To further understand the biological functions of TRIM44 in breast cancer, we screened TRIM44 target genes by performing microarray analysis for MDA-MB-231 cells treated with siTRIM44 or siControl. The top 20 up- and downregulated RefSeq transcripts in comparison with gene expression in siTRIM44-B versus siControl-B are listed in Tables S1 and S2. Among the candidate TRIM44 target genes based on the data, we examined several mRNA expressions for genes of interest by qRT–PCR. Notably, upregulation of the tumor-suppressor cyclin-dependent kinase 19 (*CDK19*) gene was shown in both MCF-7 and MDA-MB-231 cells transfected with either siTRIM44-A or -B (Figure 4A). In regard to the matrix metallopeptidase 1 (*MMP1*) gene, downregulation was shown in MDA-MB-231 cells transfected with either siTRIM44-A or -B (Figure 4B). *MMP1* expression was not clearly detected in MCF-7 cells, as the mRNA level was very low. We further investigated whether NF-κB signaling modulates the expression of potential TRIM44 target genes in breast cancer cells. Although the mRNA expression of *CDK19* was marginally affected by TNFα (0.85-fold), *MMP1* expression was significantly induced by TNFα in MDA-MB-231 cells (Figure 4C). These results suggest that the expression of some TRIM44 target genes such as *MMP1* could be possibly regulated through NF-κB signaling.

## 3. Discussion

In the present study, we demonstrated that high TRIM44 immunoreactivity (IR) is an independent poor prognostic factor in patients with breast cancer. TRIM44 IR is also positively associated with nuclear grade (NG), which is assumed to be a poor prognostic factor that represents proliferative activity of cancer cells and nuclear atypia [25,26]. Consistent with clinicopathological observations, in vitro experiments in the present study showed that TRIM44 promotes cell proliferation or migration. Furthermore, we revealed that TRIM44 modulates the expression of some TRIM44-regulated genes, along with activation of the NF-κB pathway. Taken as a whole, this study indicates that TRIM44 has distinct relevance in the pathophysiology of breast cancer.

It has been reported that the TRIM44 gene locus is frequently amplified in epithelial cancers, including breast cancer, and that TRIM44-amplified cancers likely have poorer outcomes. Based on the breast cancer cohort of the METABRIC database, ~6% of the patients had TRIM44 amplifications or gains and had poorer prognosis than patients with normal copy number [21]. Their findings are in line with the finding from the TCGA dataset of higher expression of TRIM44 mRNA in invasive ductal breast cancers compared with that in normal breast tissues. The immunohistochemical study further demonstrated that the frequency of tumors with strong TRIM44 IR seems to be much higher than that of tumors with TRIM44 gene amplifications or gains, suggesting some upregulatory mechanisms of TRIM44, other than gene gains.

The microarray analysis provided us useful information in terms of candidate transcripts regulated by TRIM44. As validated by qRT–PCR, we showed that cyclin-dependent kinase 19 (*CDK19*) and matrix metallopeptidase 1 (*MMP1*) could be upregulated and downregulated by TRIM44 knockdown, respectively. Cyclin-dependent kinases are proteins that exert their enzymatic activity by interacting with cyclin proteins [27]. Among them, CDK19 was identified as a mediator-related cyclin-dependent kinase that may be involved in transcriptional regulation [28]. Indeed, CDK19 has been shown to phosphorylate the intracellular domain of notch 1 together with cyclin C [29]. Because the cyclin C (*CCNC*) gene is often deleted in malignancies such as acute lymphoblastic leukemia [30] and osteosarcoma [31], CCNC and CDK19 may function as tumor suppressors. Interestingly, we showed that *CDK19* is upregulated by TRIM44 siRNAs in a pluripotent human testicular embryonic cancer cell line [23], suggesting that TRIM44 also plays a tumor-promoting role in the pathogenesis of other types of cancer by modulating *CDK19* expression. Meanwhile, it was reported that high expression of *CDK19* is associated with poor prognosis of breast cancer patients [32] with enhancement of estrogen receptor signaling [33]. Further functional investigation on the mechanism of transcriptional regulation by CDK19 in breast cancer cells would be required to clarify the apparently opposite roles of CDK19 among different studies. MMP1 belongs to the metalloproteinase family and has been implicated in the progression and metastasis of breast cancer [34,35,36,37,38]. In a migration assay, we found that TRIM44 affected the motility of breast cancer cells. Together with increasing production of MMP1 and enhancing motility, TRIM44 may promote tumor invasion and metastasis. It is tempting to speculate that this might be another potential mechanism for the unfavorable prognostic effect of TRIM44.

Recent studies suggest that TRIM-family proteins play critical roles in the regulation of the nuclear factor-kappa B (NF-κB)-dependent pathway by modulating ubiquitination of its signaling proteins at different steps [39,40]. In terms of TRIM44, activation of the NF-κB signaling pathway has been reported in lung cancer cells [18] and hepatic cancer cells [22]. Of note, NF-κB-dependent transcriptional activity is also associated with poor prognosis for breast cancer patients [41,42,43,44]. The present study further showed that TRIM44 is closely related to the NF-κB pathway in breast cancer cells, as exemplified by TRIM44 knockdown-mediated repression of p65 or IκBα phosphorylation. We also showed that a putative TRIM44 target gene, *MMP1*, was significantly upregulated by the NF-κB activator TNFα in MDA-MB-231 cells, as shown in previous literature [45].

So far, the mechanism underlying the TRIM44-dependent activation of NF-κB signaling has not been well clarified in cancers. TRIM44 has a zinc-finger domain that is found in members of the ubiquitin-specific protease family [8] that removes ubiquitin and prevents substrate proteins from degradation [46]. Therefore, the function of TRIM44 may be mediated by stabilization of unknown substrates. In the context of antiviral response, TRIM44 is shown to stabilize virus-induced signaling adaptor (VISA) and enhance virus-induced NF-κB signaling [47]. It may be intriguing to investigate whether TRIM44 also stabilizes molecules similar to VISA in breast cancer cells. We previously showed that TRIM44 stabilizes another TRIM family member, TRIM17 [48]. Therefore, it is possible that the function of TRIM44 is partially attributable to that of TRIM17 and its substrates, such as the ZW10-interacting protein (ZWINT) [49]. Meanwhile, cultured cell experiments revealed that mTOR signaling could be one of the major enriched pathways in gastric cancer cells with TRIM44 overexpression [21]. In prostate cancer cells, Akt-dependent regulation of NF-κB is controlled by mTOR and Raptor, in association with IKK [50]. Notably, it was also reported that knockdown of TRIM44 suppressed PI3K/Akt signaling in a prostate cancer cell line [51]. Therefore, it is possible that the PI3K/Akt/mTOR pathway might be involved in the TRIM44-dependent NF-κB activation in breast cancer cells.

In the present study, only thirteen patients with ER-negative breast cancer were included for analysis. Nevertheless, our in vitro study using an ER-negative cell line, MDA-MB-231, suggested TRIM44 affected malignant characters of ER-negative breast cancer cells. It should be important to include an increased number of patients with ER-negative breast cancer in future studies to evaluate the prognostic and therapeutic value of TRIM44 immunoreactivity on ER-negative cancer patients. It would be interesting if we could also evaluate BRCA 1/2 and Ki-67 statuses, and their relationship with ER and TRIM44 statuses.

## 4. Materials and Methods

### 4.1. Collection of Human Tissue Samples and Clinical Data

Tissue samples of invasive breast cancer were obtained from 129 Japanese female breast cancer patients who underwent surgical treatment from 2006 to 2013 at Toranomon Hospital, Tokyo, Japan (range of age, 31–81 years). No patients received chemotherapy or molecular-target therapy before surgery. Standard adjuvant treatments were selected according to the clinical practice guidelines of the National Comprehensive Cancer Network [52]. Staging was performed according to ‘TNM classification of Malignant Tumours’ (7th Edition, 2009). The clinical outcome was evaluated by distant disease-free survival and overall survival in this study. Distant disease-free survival was defined as time span from date of surgery to the first distant recurrence or last follow-up. Similarly, overall survival was defined as time from date of surgical therapy to death or last follow-up. The mean follow-up duration was 81 months (range, 8–118 months) in this study. This study was approved by the ethical committee in Toranomon Hospital (approval number: 845) and Saitama Medical University (approval number: 13-148). All the patients provided written informed consent to participate in this study.

### 4.2. Antibodies

Rabbit monoclonal antibodies for estrogen receptor alpha (clone: SP1), progesterone receptor (clone: 1E2) and human epidermal growth factor receptor 2 (HER2) (clone: 4B5) were purchased from Roche (Basel, Switzerland). Mouse monoclonal β-Actin antibody (clone: AC-74) was purchased from Sigma (St. Louis, MO, USA). Rabbit monoclonal nuclear factor-kappa B (NF-κB) p65 subunit antibody (clone: D14E12), rabbit monoclonal phospho-NF-κB p65 (Ser536) antibody (clone: 93H1), mouse monoclonal NF-κB inhibitor alpha (IκBα) antibody (clone: L35A5) and rabbit monoclonal phospho-IκBα (Ser32) antibody (clone: 14D4) were obtained from Cell Signaling Technology (Beverly, MA, USA). Anti-TRIM44 polyclonal antibody was raised in our laboratory [23]. Briefly, rabbits were immunized with a glutathione S-transferase (GST) fusion protein with amino acids of full-length mouse TRIM44 protein as an antigen. Anti-GST antibody was removed from serum using GST-bound resin. The serum was further purified by affinity to mouse TRIM44 antigen. The cross-reactivity to human TRIM44 protein was confirmed by western blotting using overexpressed human TRIM44 [23] and siTRIM44 (Figure 2A).

### 4.3. Immunohistochemistry

Immunohistochemical analysis of TRIM44 expression was performed using an EnVision + visualization kit (Dako, Carpinteria, CA, USA) as previously described [53]. The tissue sections (6 μm) were deparaffinized, rehydrated through a graded ethanol series and rinsed in Tris-buffered saline containing 0.05% Tween-20 (TBST). For antigen retrieval, the sections were heated in a water bath at 100 °C for 60 min in a 10 mM sodium citrate buffer at pH 6.0. After the endogenous peroxidase activity was blocked with 0.3% H_2_O_2_, the sections were incubated in 10% fetal bovine serum for 30 min. The primary antibody, a polyclonal antibody against TRIM44 (1:50 dilution), was applied, and the samples were incubated overnight at 4 °C. The sections were rinsed in TBST and incubated with EnVision + horseradish peroxidase-labeled polymer for 1 h at room temperature. The antigen–antibody complex was visualized using a DAB (3,3′-diaminobenzidine) substrate kit for peroxidase (Vector Laboratories, Burlingame, CA, USA). Rabbit immunoglobulin G was used in place of the primary antibody as a negative control.

Immunostained slides were evaluated for intensity scores and proportional scores. Intensity scores of immunostaining were rated from 0 to 3+ (0: none, 1: weak, 2: moderate, 3: strong) and proportional scores were defined by percentage of stained tumor cells. Immunoreactivity (IR) was defined as “strong” when over 1/3 of tumor cells were stained as 2+ or 3+. Two observers (K.K. and H.K.) evaluated the slides, and the third observer (T.F.) estimated the IR in case of disagreement between the 2 observers.

Immunostaining of estrogen receptor alpha (ER), progesterone receptor (PgR), and HER2 was performed automatically using Ventana BenchMark GX (Roche). ER and PgR statuses were judged positive when nuclear staining of more than 1% of the tumor cells was observed according to the guidelines of the American Society of Clinical Oncology/College of American Pathologists (ASCO/CAP) [54]. HER2 status was judged by the updated guideline from ASCO/CAP [55].

### 4.4. Cell Culture

Human breast cancer cell line MDA-MB-231 was obtained from ATCC (Manassas, VA, USA) and MCF-7 cell line was obtained from RIKEN Cell Bank (Tsukuba, Japan). Cell line authentication was performed using Gene Print 10 System (Promega, Madison, WI, USA). The short tandem repeat profiles were completely matched with the registered database of ATCC in both MDA-MB-231 and MCF-7 cells. MCF-7 cell line was cultured in Dulbecco’s modified Eagle’s medium (DMEM) at 37 °C with 5% CO_2_ and MDA-MB 231 cell line was cultured in Leibovitz’s L15 medium without CO_2_. DMEM was purchased from Sigma-Aldrich Japan (Tokyo, Japan) and Leibovitz’s L15 medium was purchased from Wako Pure Chemical Industries (Osaka, Japan). They were used with 10% fetal bovine serum (FBS) and 1% penicillin–streptomycin (Wako Pure Chemical Industries).

### 4.5. Plasmid Construction and Transfection

Construction of human TRIM44 cDNA was previously described [23]. Briefly, Flag-tagged human TRIM44 was subcloned into the mammalian expression vector, pcDNA3. Transfection of expression vectors was performed 24 h after seeding cells using FuGENE HD (Promega), according to the manufacturer’s instructions.

### 4.6. Small Interfering RNA Transfection

Knocking down the expression of TRIM44 was carried out using small interfering RNA (siRNA) transfection. Two specific siRNAs targeting TRIM44 (siTRIM44-A and siTRIM44-B), and two control siRNA not targeting human transcripts (siControl-A and siControl-B), were purchased from RNAi Inc (Tokyo, Japan). siControl-A is a siRNA targeting firefly luciferase, and siControl-B is a siRNA without specific targets. These siRNAs were transfected into breast cancer cells (MCF-7 and MDA-MB-231) at the time of seeding cells by a reverse-transfection method using Lipofectamine RNAiMAX (Invitogen, St. Louis, MO, USA) according to the manufacture’s instruction at the indicated concentrations. The sequences of siRNA were as follows.
siControl-A (siLuciferase)Sense: 5′-GUGGAUUUCGAGUCGUCUUAA-3′Anti-sense: 5′-AAGACGACUCGAAAUCCACAU-3′siControl-BSense: 5′-GUACCGCACGUCAUUCGUAUC-3′Anti-sense: 5′-UACGAAUGACGUGCGGUACGU-3′siTRIM44-ASense: 5′-CCGAGUAAGCAGGGAUGUACU-3′Anti-sense: 5′-UACAUCCCUGCUUACUCGGGA-3′siTRIM44-BSense: 5′-GAAUCAGUCGGAUACUCAUAG-3′Anti-sense: 5′-AUGAGUAUCCGACUGAUUCUG-3′

### 4.7. Western Blot Analysis

Whole-cell lysates were prepared using PLC lysis buffer containing 1% Triton X-100 [56]. They were separated on 10% sodium dodecyl sulfate polyacrylamide gel electrophoresis (SDS–PAGE), and then transferred to polyvinylidene difluoride (PVDF) membranes (Millipore, Bedford, MA, USA). The membranes were blocked in Blocking One (Nacalai Tesque, Kyoto, Japan) for 30 min. The membranes were incubated with primary antibodies, followed by incubation with horseradish peroxidase (HRP)-conjugated secondary antibody (GE Healthcare, Buckinghamshire, UK). The bound antibodies were visualized with ECL Prime Western Blotting Detection Reagent (GE Healthcare). When NF-κB signaling was evaluated, MCF-7 cells or MDA-MB-31 cells were treated with TNFα (PeproTech, Rocky Hill, NJ, USA) at the indicated concentration or vehicle (PBS) for 5 min before cell lysis.

### 4.8. Cell Proliferation Assay

Cell proliferation assay was performed using 96-well plates. On the first day, the cells were seeded at the concentration of 5.0 × 10^3^ cells/well (MCF-7) or 1.0 × 10^4^ cells/well (MDA-MB-231) with indicated siRNAs (10 nM) in a reverse-transfection method. Cell growth was evaluated on the 5th day after transfection by MTS assay using The Cell Titer 96 Aqueous One Solution Cell Proliferation Assay (Promega) according to the manufacturer’s instructions. Assays were performed in quadruplicate, and data are presented as mean value ± SEM.

### 4.9. Cell Migration Assay

Cell migration assay was performed as previously described [56]. Briefly, cell culture inserts with polyethylene terephthalate (PET) filters containing 8.0 μm pores in the 24-well-plate format (BD Bioscience, Bedford, MA, USA) were used for the assay. The lower surface of the membrane was pre-treated with 10 μg/mL fibronectin (Sigma) for 30 min. MDA-MB-231 cells transfected with siTRIM44 or siControl (10 nM) 24 h before the assay were added to the upper chambers, and allowed to migrate for another 24 h. Then, the filters were dipped in methanol for 30 min, washed with PBS and stained with Giemsa stain solution (Muto Pure Chemicals, Tokyo, Japan) for 30 s. After washing 3 times with PBS, filters were mounted on glass slides. The cells migrated on the lower surface were counted in five randomly selected fields under a microscope at a magnification of ×400. Data are presented as mean value ± SEM.

### 4.10. Luciferase Assay

MCF-7 or MDA-MB-231 cells were transfected with NF-κB luciferase reporter plasmid (pNFκB-Luc, Stratagene, La Jolla, CA, USA) using FuGENE HD (Promega). When necessary, TRIM44 expression vector or empty vector (pcDNA3) was transfected at the same time. In the experiment using siRNAs, the cells were detached from the dish and transfected with indicated siRNAs (2 nM) by a reverse-transfection method. After 48 h, cells were treated at the indicated concentrations of TNFα (PeproTech) for 5 h. Cell lysate was analyzed by Luciferase Assay System (Promega) according to the manufacturer’s protocol. Luciferase activities normalized with the protein concentration measured by BCA assay (Thermo Fisher Scientific, Waltham, MA, USA) were shown as relative luciferase units (R.L.U.).

### 4.11. Microarray Analysis

Transfection of siTRIM44-B or siControl-B (2 nM) was performed by a reverse-transfection method. Total RNAs from MDA-MB-231 cells transfected with siRNAs were extracted by using an RNA extraction kit (Isogen, Nippon Gene, Tokyo, Japan) according to the manufacturer’s instructions 48 h after transfection of siRNAs. RNA integrity number (RIN) values were above 9.0 in all RNA samples. The GeneChip Human Gene 1.0 ST array (Affymetrix, Santa Clara, CA, USA) was used according to the manufacture’s protocol. Transcripts whose expression levels were below −1SD in both treatments were excluded from further analyses. Fold changes of gene expressions were log2 transformed.

### 4.12. Quantitative Reverse Transcription—Polymerase Chain Reaction

Quantitative reverse transcription polymerase chain reaction (qRT–PCR) was performed as previously described with some modification [57]. Briefly, total RNAs were extracted from cells prepared in biological triplicate using ISOGEN (Nippon Gene), followed by cDNA synthesis using PrimeScript (Takara, Kyoto, Japan). The cDNA was subjected to real-time polymerase chain reaction (PCR) using a Fast 7500 real-time PCR system (Applied Biosystems, Foster City, CA, USA) based on detection of SYBR Green fluorescence (Kapa Biosystems, Woburn, MA, USA). mRNA expression levels were normalized with *GAPDH* by the 2^−ΔΔ*C*t^ method [58]. Sequences of primers are as follows.
*GAPDH* forward: 5′-TCTAGTAAAGTGGATATTGTTG-3′*GAPDH* reverse: 5′-GATGGTGATGGGATTTCC-3′*CDK19* forward: 5′-GAGCATGACTTGTGGCATATT-3′*CDK19* reverse: 5′-TGGATACCATCAAGAATCTGGT-3′*MMP1* forward: 5′-CGCACAAATCCCTTCTACCC-3′*MMP1* reverse: 5′-AACAGCCCAGTACTTATTCCCT-3′

### 4.13. Statistical Analyses

Statistical analyses were conducted using JMP^®^ 11.0.2 (SAS Institute, Cary, NC, USA) or Excel Statistics 2010 (add-in software for Microsoft Excel) (SSRI, Tokyo, Japan). In the immunohistochemical analyses, Student’s *t*-test or Pearson’s chi-square (χ^2^) test were used to evaluate the relation between TRIM44 immunoreactivity and clinicopathological parameters. Distant disease-free survival and overall survival curves were generated by the Kaplan–Meier method. Then, statistical significance was calculated using the log-rank test. Univariate and multivariate analyses were evaluated by a logistic regression model and Cox proportional hazard model, respectively. In the in vitro experiments, the statistical analyses were performed using Student’s *t*-test, one-way ANOVA followed by Dunnett’s test as a post-hoc analysis, or two-way ANOVA.

## 5. Conclusions

Our clinical study demonstrated that breast cancer patient outcomes are correlated with the immunoreactivity detected by the anti-TRIM44 antibody, which could be used as a potential biomarker to predict poor prognosis for survival of patients. Functional studies suggested that the augmented NF-κB signaling pathway would be the underlying mechanism of TRIM44 function. TRIM44 knockdown caused attenuated proliferation and migration of breast cancer cells, suggesting that TRIM44 may be a potential therapeutic target for breast cancer. These findings provide new clues to develop alternative effective strategies for breast cancer management.

## Figures and Tables

**Table 1:** Relationship between the TRIM44 immunoreactivity and clinicopathological parameters in 129 breast cancer patients. ER, estrogen receptor; PgR, progesterone receptor; HER2, human epidermal growth factor receptor 2; IR, immunoreactivity; SEM, standard error of the mean.

	TRIM44 Status		*p*-Value
	Weak IR (*n* = 62)	Strong IR (*n* = 67)	
**Age (±SEM)**	55.6 ± 1.4	52.2 ± 1.3	0.044
≤50 years old	22	34	0.081
>50 years old	40	33	
**Stage**			0.933
I	31	33	
II, III	31	34	
**Pathological tumor size**			0.476
≤20 mm	36	43	
>20 mm	26	24	
**Lymph node status**			0.131
Negative	44	39	
Positive	18	28	
**ER status**			0.305
Negative	8	5	
Positive	54	62	
**PgR status**			0.201
Negative	18	13	
Positive	44	54	
**HER2 status**			0.353
Negative	51	59	
Positive	11	8	
**Nuclear grade**			0.033
1	42	33	
2, 3	20	34	

**Table 2:** Univariate and multivariate analyses of clinicopathological parameters and distant disease-free survival in 129 breast cancer patients. CI, confidence interval; ER, estrogen receptor; PgR, progesterone receptor; HER2, human epidermal growth factor receptor 2; IR, immunoreactivity.

Variables	Univariate	Multivariate		
	*p*-Value	Hazard Ratio	95% CI	*p*-Value
**Distant disease-free survival**				
Age (>50 vs. ≤50 years old)	0.0873			
Tumor size (>20 vs. ≤20 mm)	**0.0019**	4.58	1.89–12.2	**0.0007**
Lymph node status (+/−)	0.0583			
ER status (−/+)	**0.0072**	4.14	1.40–10.9	**0.0124**
PgR status (−/+)	0.4030			
HER2 status (+/−)	0.8000			
Nuclear grade (2, 3/1)	**0.0019**	2.58	1.03–7.36	**0.0430**
TRIM44 IR (strong/weak)	**0.0281**	3.86	1.48–11.5	**0.0051**
**Overall survival**				
Age (>50 vs. ≤50 years old)	0.2272			
Tumor size (>20 vs. ≤20 mm)	**0.0018**	9.41	2.77–43.8	**0.0002**
Lymph node status (+/−)	0.2864			
ER status (−/+)	**0.0059**	6.82	1.85–22.4	**0.0052**
PgR status (−/+)	0.1455			
HER2 status (+/−)	0.8319			
Nuclear grade (2, 3/1)	**0.0004**	7.30	1.93–47.6	**0.0002**
TRIM44 IR (strong/weak)	**0.0223**	8.11	2.09–47.7	**0.0017**

Significant *p*-values were expressed in bold type.

## Supplementary Materials

Supplementary materials can be found at www.mdpi.com/1422-0067/18/9/1931/s1.
