# Supplementary material for: TRIM44 Is a Poor Prognostic Factor for Breast Cancer Patients as a Modulator of NF-κB Signaling
Source: Int J Mol Sci. 2017 Sep 8;18(9):1931. doi: 10.3390/ijms18091931 (PMC5618580; doi:10.3390/ijms18091931)
Supplement: Supplementary file 1 [file ijms-18-01931-s001.pdf]

Table S1. Top 20 genes up-regulated by TRIM44 knockdown in MDA-MB-231 cells.

	Gene Symbol	Description	Fold change
1	C6orf99	chromosome 6 open reading frame 99 (C6orf99), mRNA	2.178
2	CDK19	cyclin-dependent kinase 19 (CDK19), mRNA	2.114
3	ZNF204P	zinc finger protein 204, pseudogene (ZNF204P), transcript variant 1, non-coding RNA	1.964
4	FAM231A	family with sequence similarity 231, member A (FAM231A), mRNA	1.922
5	ZNF594	zinc finger protein 594 (ZNF594), mRNA	1.900
6	C7orf69	chromosome 7 open reading frame 69 (C7orf69), mRNA	1.878
7	TMEM229B	transmembrane protein 229B (TMEM229B), mRNA	1.756
8	IGLON5	IgLON family member 5 (IGLON5), mRNA	1.748
9	MIR29A	microRNA 29a (MIR29A), microRNA	1.737
10	RNU6-23P	RNA, U6 small nuclear 23, pseudogene (RNU6-23P), small nuclear RNA	1.720
11	LGALS9C	lectin, galactoside-binding, soluble, 9C (LGALS9C), mRNA	1.702
12	CEACAM8	carcinoembryonic antigen-related cell adhesion molecule 8 (CEACAM8), mRNA	1.674
13	LRRC17	leucine rich repeat containing 17 (LRRC17), transcript variant 1, mRNA	1.661
14	KU-MEL-3	uncharacterized LOC497048 (KU-MEL-3), long non-coding RNA	1.654
15	PLSCR4	phospholipid scramblase 4 (PLSCR4), transcript variant 1, mRNA	1.642
16	SAA2	serum amyloid A2 (SAA2), mRNA	1.639
17	FABP6	fatty acid binding protein 6, ileal (FABP6), transcript variant 1, mRNA	1.638
18	CDRT1	CMT1A duplicated region transcript 1 (CDRT1), transcript variant 2, mRNA	1.618
19	DNAH6	dynein, axonemal, heavy chain 6 (DNAH6), mRNA	1.604
20	OR2B6	olfactory receptor, family 2, subfamily B, member 6 (OR2B6), mRNA	1.604

Table S2. Top 20 genes down-regulated by TRIM44 knockdown in MDA-MB-231 cells.

	Gene Symbol	Description	Fold change
1	TRIM44	tripartite motif containing 44 (TRIM44), mRNA	0.3747
2	G6PD	glucose-6-phosphate dehydrogenase (G6PD), transcript variant 1, mRNA	0.3871
3	CYBRD1	cytochrome b reductase 1 (CYBRD1), transcript variant 2, mRNA	0.4618
4	EML4	echinoderm microtubule associated protein like 4 (EML4), transcript variant 2, mRNA	0.4638
5	SORD	sorbitol dehydrogenase (SORD), transcript variant 1, mRNA	0.4708
6	GXYLT1	glucoside xylosyltransferase 1 (GXYLT1), transcript variant 2, mRNA	0.4765
7	TLR4	toll-like receptor 4 (TLR4), transcript variant 3, mRNA	0.4828
8	CT55	cancer/testis antigen 55 (CT55), transcript variant 1, mRNA	0.5366
9	MCM9	minichromosome maintenance complex component 9 (MCM9), transcript variant 1, mRNA	0.5435
10	PRTFDC1	phosphoribosyl transferase domain containing 1 (PRTFDC1), transcript variant 2, mRNA	0.5482
11	GPR63	G protein-coupled receptor 63 (GPR63), transcript variant 1, mRNA	0.5555
12	MTOR	mechanistic target of rapamycin (serine/threonine kinase) (MTOR), mRNA	0.5575
13	PCDHB8	protocadherin beta 8 (PCDHB8), mRNA	0.5670
14	UBLCP1	ubiquitin-like domain containing CTD phosphatase 1 (UBLCP1), mRNA	0.6594
15	MMP1	matrix metallopeptidase 1 (interstitial collagenase) (MMP1), transcript variant 2, mRNA	0.5729
16	SLC29A4	solute carrier family 29 (equilibrative nucleoside transporter), member 4 (SLC29A4), transcript variant 1, mRNA	0.5795
17	PRSS2	protease, serine, 2 (trypsin 2) (PRSS2), mRNA	0.5840
18	FAM183A	family with sequence similarity 183, member A (FAM183A), mRNA	0.5888
19	PLEKHS1	pleckstrin homology domain containing, family S member 1 (PLEKHS1), transcript variant 2, mRNA	0.5906
20	ZADH2	zinc binding alcohol dehydrogenase domain containing 2 (ZADH2), mRNA	0.6051

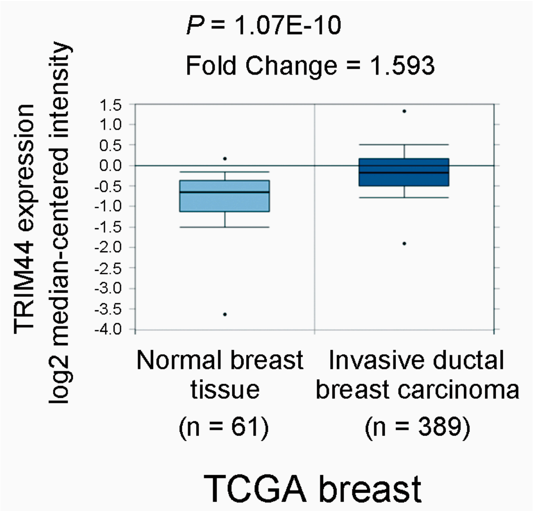

Figure S1. Box-whisker plots showing differential mRNA expression of TRIM44 in cancerous and non-cancerous breast tissues. The horizontal bars designate median values, and the boxes indicate the upper and the lower quartiles. The whiskers show the 90% range of values and the plots represent the maximus and the minimum values. TRIM44 mRNA expression in invasive ductal breast cancer is higher compared with normal breast tissues (Oncomine data sets: TCGA Breast).

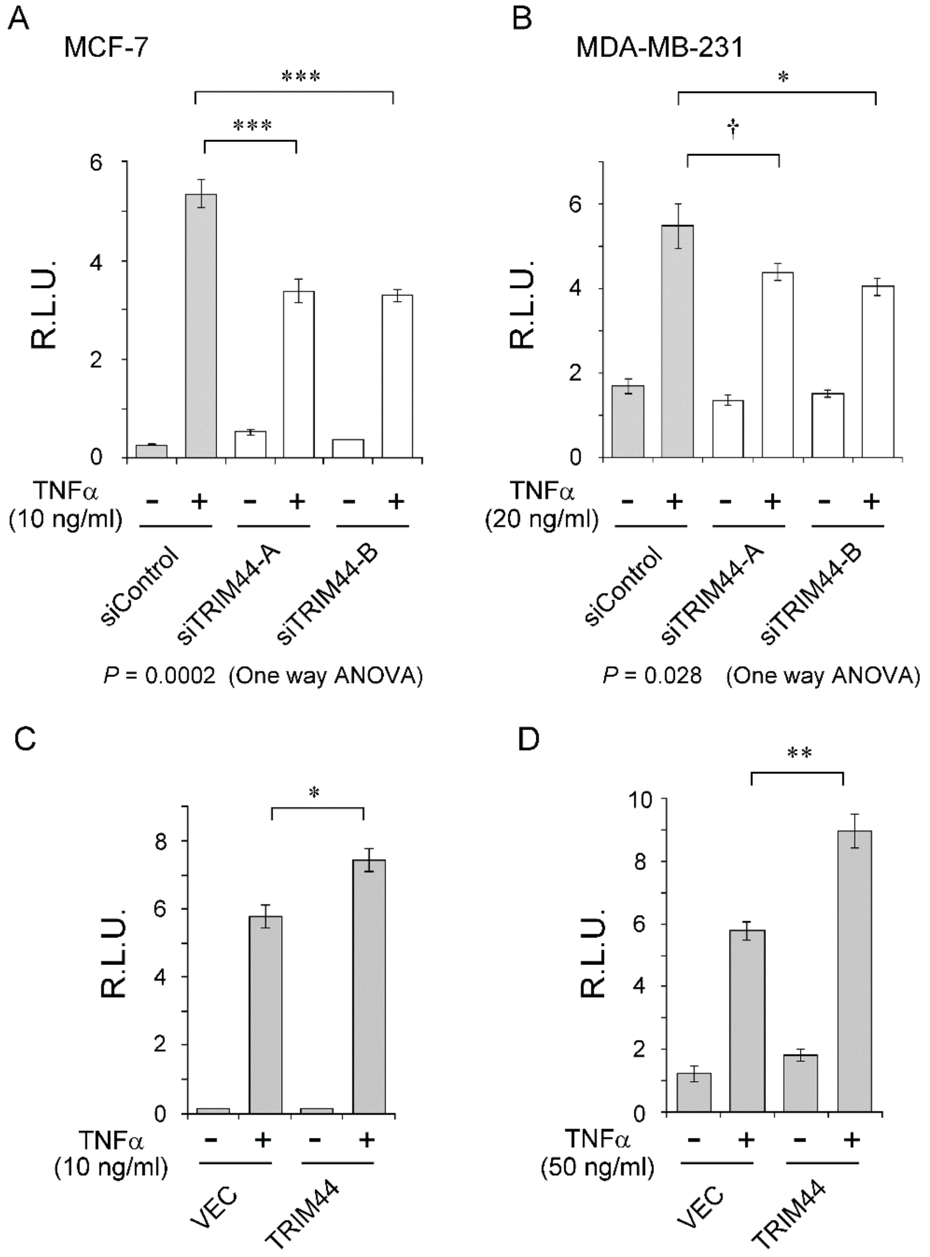

Figure S2. TRIM44 knockdown impaired NF-κB transcriptional activity in breast cancer cells. (**A** and **B**) MCF-7 cells or MDA-MB-231 cells were transfected with NF-κB reporter plasmid. On the next day, the cells were detached from the dish and indicated siRNAs (2 nM) were transfected by reverse transcription method. siControl-B was used as a negative control. Forty-eight hours after siRNA transfection, cells were treated with indicated concentrations of TNF*α* or vehicle (phosphate buffered saline) for another 5 h before measuring luciferase activities. Luciferase activities were shown as relative luciferase units (R.L.U.). Results are expressed as mean ± SEM (n = 6). R.L.U. values of TNF*α* stimulated group analyzed by one-way ANOVA were significantly different in both MCF-7 and MDA-MB-231 cells. Post-hoc Dunnett's test was performed in comparison with siControl group. † $P < 0.1$, * $P < 0.05$, *** $P < 0.001$. (**C** and **D**) TRIM44 expression vector (TRIM44) or empty vector (VEC) were transfected to MCF-7 cells or MDA-MB-231 cells together with NF-κB reporter plasmid. Twenty four hours after transfection, cells were treated with indicated concentrations of TNF*α* or vehicle (phosphate buffered saline) for another 5 hours before measuring luciferase activities. Results are expressed as mean ± SEM (n = 3), * $P < 0.05$, ** $P < 0.01$ (*t*-test).
